# Maximal-Effort Cytoreductive Surgery for Ovarian Cancer Patients with a High Tumor Burden: Variations in Practice and Impact on Outcome

**DOI:** 10.1245/s10434-019-07516-3

**Published:** 2019-06-26

**Authors:** Marcia Hall, Konstantinos Savvatis, Katherine Nixon, Maria Kyrgiou, Kuhan Hariharan, Malcolm Padwick, Owen Owens, Paula Cunnea, Jeremy Campbell, Alan Farthing, Richard Stumpfle, Ignacio Vazquez, Neale Watson, Jonathan Krell, Hani Gabra, Gordon Rustin, Christina Fotopoulou

**Affiliations:** 1 Mount Vernon Cancer Centre, Northwood, Middlesex UK; 2 Inherited Cardiovascular Diseases Unit, Barts Heart Centre, London, UK; 3 William Harvey Research Institute, Queen Mary University, London, UK; 4 Department of Surgery and Cancer, Imperial College London and West London Gynecological Cancer Centre, Imperial College NHS Trust, London, UK; 5 West Hertfordshire Gynaecological Cancer Centre, WHH NHS Trust, Watford, UK; 6 Department of Anaesthetics, Centre for Perioperative Medicine and Critical Care Research, Imperial College Healthcare NHS Trust, Ham House, Hammersmith Hospital, London, UK; 7 Department of Gynaecology, Hillingdon Hospital, Pield Heath Road, Uxbridge, UK; 8 Early Clinical Development, IMED Biotech Unit, AstraZeneca, Cambridge, UK

## Abstract

**Background:**

This study aimed to compare the outcomes of two distinct patient populations treated within two neighboring UK cancer centers (A and B) for advanced epithelial ovarian cancer (EOC).

**Methods:**

A retrospective analysis of all new stages 3 and 4 EOC patients treated between January 2013 and December 2014 was performed. The Mayo Clinic surgical complexity score (SCS) was applied. Cox regression analysis identified the impact of treatment methods on survival.

**Results:**

The study identified 249 patients (127 at center A and 122 in centre B) without significant differences in International Federation of Gynecology and Obstetrics (FIGO) stage (FIGO 4, 29.7% at centers A and B), Eastern Cooperative Oncology Group (ECOG) performance status (ECOG < 2, 89.9% at centers A and B), or histology (serous type in 84.1% at centers A and B). The patients at center A were more likely to undergo surgery (87% vs 59.8%; *p* < 0.001). The types of chemotherapy and the patients receiving palliative treatment alone were equivalent between the two centers (3.6%). The median SCS was significantly higher at center A (9 vs 2; *p* < 0.001) with greater tumor burden (9 vs 6 abdominal fields involved; *p* < 0.001), longer median operation times (285 vs 155 min; *p* < 0.001), and longer hospital stays (9 vs 6 days; *p* < 0.001), but surgical morbidity and mortality were equivalent. The independent predictors of reduced overall survival (OS) were non-serous histology (hazard ratio [HR], 1.6; 95% confidence interval [CI] 1.04–2.61), ECOG higher than 2 (HR, 1.9; 95% CI 1.15–3.13), and palliation alone (HR, 3.43; 95% CI 1.51–7.81). Cytoreduction, of any timing, had an independent protective impact on OS compared with chemotherapy alone (HR, 0.31 for interval surgery and 0.39 for primary surgery), even after adjustment for other prognostic factors.

**Conclusions:**

Incorporating surgery into the initial EOC management, even for those patients with a greater tumor burden and more disseminated disease, may require more complex procedures and more resources in terms of theater time and hospital stay, but seems to be associated with a significant prolongation of the patients overall survival compared with chemotherapy alone.

Maximal-effort cytoreductive surgery aimed at total macroscopic tumor clearance combined with platinum-based chemotherapy and targeted agents is the cornerstone of modern primary epithelial ovarian cancer (EOC) management.^1^ Although findings have shown high tumor burden to be associated with a less favorable overall outcome than more advantageous tumor dissemination patterns with less disease,^2^ multiple prospective and retrospective series have long demonstrated a strong positive association between total macroscopic tumor clearance rates and survival, not only on an individual basis but also at the level of large patient cohorts, in which individual tumor biology-related factors are less likely to skew collective survival data.^1^,^3^–^8^

The team of Chi et al. recently presented the survival data for all advanced EOC patients treated at Memorial Sloan Kettering categorized by year of primary debulking surgery based on the implementation of surgical changes in their approach to ovarian cancer debulking. Their study demonstrated that complete gross resection rates, progression-free survival (PFS) and overall survival (OS) increased during the 13-year evaluation period despite operating on higher-stage disease and patients with a greater tumor burden. This was assumed to be largely attributable to the surgical paradigm shifts implemented specifically to achieve more complete surgical cytoreduction, even for patients with a less favorable disease profile.^4^

Nevertheless, as with all medical and surgical advances, their broader implementation varies greatly nationally and internationally, not just due to differences in the available resources, but also because of long-established local practice and broad disparities in overall philosophy as well as in individual and infrastructural expertise.^3^,^6^,^8^,^9^ Especially for patients with a high tumor burden, in which therapeutic effort often is challenged, not only by the disease itself but also by the impact that this advanced disease has on the actual patient, both personal and infrastructural resources and expertise often are stretched, and hence reasonable doubt arises about the limitations and limits of optimal treatment.^2^,^3^,^6^

The current analysis aimed to demonstrate how differences in local practice may influence the patient’s outcome by evaluating not only the surgical patients, but also the entire EOC cohort treated at one of two large UK cancer centers in an attempt to exclude a selection bias of seemingly more favorable and operable patients^7^,^10^,^11^ and have all ovarian cancer patients in the denominator, including those women with more adverse tumor profiles and higher tumor load.

## Materials and Methods

After local ethical board approvals were obtained, a retrospective comparative audit was performed to evaluate types of medical and surgical treatments as well as tumor dissemination patterns and survival for all consecutive patients with newly diagnosed stages 3 and 4 EOC treated between January 2013 and December 2014 at two neighboring cancer centers (A and B). Patients with relapsed disease or non-epithelial histologies were excluded.

**Fig. 1:**
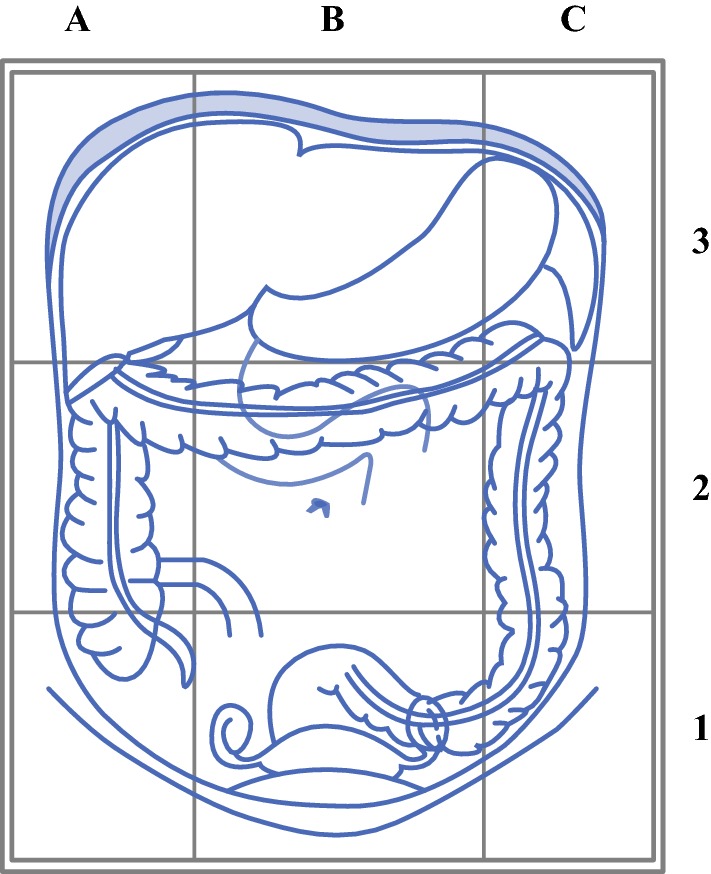
The Intraoperative Mapping of Ovarian Cancer tool divides the abdomen into nine fields, three at each abdominal level as follows: lower (level 1), middle (level 2), and upper (level 3) abdomen. Scoring is performed by allocating + 1 for each field in which cancer is visible

Patient- and tumor-related characteristics, surgical findings and procedures, tumor dissemination patterns, and morbidity were retrospectively retrieved from patients’ notes. Surgical complexity scores (SCS) were calculated using the validated surgical complexity scoring system as defined by the Mayo Clinic.^8^ Intraoperative tumor dissemination patterns and tumor burden were described from the operation notes for each surgical patient using a well-established system developed and validated to obtain an objective and reproducible documentation of ovarian cancer spread (Intraoperative Mapping of Ovarian Cancer [IMO]; Fig. 1).^6^,^12^

To ensure that all patients were included, the evaluated populations were identified from the Multidisciplinary Tumorboard Meetings (MDT) of each center. Inclusion in the MDT is mandatory for every cancer patient in the National Health Service (NHS), even those who do not receive surgery, resulting in a complete capture and documentation of the entire patient population without selection bias.

### Follow-Up Evaluation

The patients were followed up every 3 months for the first 2 years and then every 6 months for 5 years in both centers as per national guidelines.^13^ However, the follow-up strategies differed between the two centers with respect to CA125 monitoring. The patients at center B were less likely to have their CA125 checked routinely based on the outcomes of the OVO5/EORTC 5595 trial.^14^ Therefore, asymptomatic relapses were more likely to be detected at center A. Given the lack of a prospective definition for relapse agreed upon by both centers, we compared only overall survival (OS) as a nonnegotiable hard end point.

### Statistical Analysis

Statistical analysis was performed using SPSS software, version 20.0 (SPSS Inc., Chicago, IL, USA). Estimates of survival were calculated using the Kaplan–Meier method. All parameters are expressed as medians with a range or means with a 95% confidence interval (95% CI). The follow-up and survival times were calculated from the day of initial treatment, chemotherapy, or surgery depending on what the patients received first, or from the date of diagnosis for the patients who received palliative treatment only.

Multivariate analysis was performed using the Cox-regression method. Continuous variables were tested for normality using the Shapiro–Wilk test, and comparisons were made with a Student’s *t* test for normally distributed variables or with the Mann–Whitney *U* test for nonparametric variables. Categorical variables were compared with Fisher’s exact test. Survival curves were compared with the log-rank test. Differences were considered significant at a *p* value lower than 0.05.

## Results

### Patient Demographics and Treatments

The study enrolled 249 patients (127 at center A and 122 at center B). Demographics and baseline tumor-related characteristics (Table 1) differed in certain aspects. The patients at center A were significantly younger by a median of 2.5 years (66.5 vs 69 years; *p *= 0.008) and had significantly higher CA125 levels. The median CA125 at center A was 667.5 U/mL compared with 461.5 U/mL at center B (*p *= 0.005). The patients at center A had lower median pretreatment albumin levels (32.5 vs 36 g/L; *p *< 0.001). The center A population was significantly more ethnically diverse, with a Caucasian population of only 65.4% compared with 95.1% at center B (*p *< 0.001). The two centers did not differ significantly in terms of stage, ECOG performance status, or histology.

**Table 1:** Demographic, tumor- and treatment-related characteristics at initial presentation for the entire patient cohorts and separately for center A versus center B

	All patients (*n *= 249) *n* (%)	Center A patients (*n *= 127) *n* (%)	Center B patients (*n *= 122) *n* (%)	*p* value
*Median age: years (range)*	67 (19–96)	66.5 (19–96)	69 years (34–94)	**0.008**
*FIGO stage*				0.423
3a	7 (2.8)	5 (3.9)	2 (1.6)	
3b	7 (2.8)	7 (5.5)	1 (0.8)	
3c	161 (64.7)	67 (52.8)	93 (76.2)^a^	
4	74 (29.7)	48 (37.8)	26 (21.3)	
*Grading*				0.067
Low grade	7 (2.8)	6 (4.7)	1 (0.8)	
High grade (G1 + G2)	242 (97.2)	121 (95.3)	121 (99.2)	
*Histology*				0.1
Serous	207 (84.1)	102 (80.3)	105 (88.2)	
Clear cell	7 (2.8)	7 (5.5)	0	
Endometrioid	7 (2.8)	1 (0.8)	6 (5)	
Mucinous	1 (0.4)	1 (0.8)	0	
Carcinosarcoma	14 (5.7)	14 (11)	0	
Mixed/other	10 (4.1)	2 (1.6)	8 (6.7)	
*Median albumin: g/L (range)*	34 (8–48)	32.5 (8–43)	36 (14–48)	**< 0.001**
*Ethnicity*				**< 0.001**
White	199 (79.9)	83 (65.4)	116 (95.1)	
Black	5 (2)	4 (3.1)	1 (0.8)	
Asian	36 (14.5)	33 (26)	3 (2.5)	
Arabic	5 (2)	5 (3.9)	0	
Chinese	4 (1.6)	2 (1.6)	2 (1.6)	
*Median CA125: U/ml range)*	554.5 (6–41,509)	667.5 (34–41,509)	461.5 (6–39,047)	**0.005**
Received surgery	184 (74)	111 (87.4)	73 (59.8)	**< 0.001**
Upfront surgery	122 (49)	98 (77.1)	24 (19.6)	**< 0.001**
Chemotherapy alone	56 (22.4)	12 (9.4)	44 (36)	**< 0.001**
Palliative treatment only	9 (3.6)	4 (3.1)	5 (4.1)	NS
Carboplatin-mono first-line	28 (11.2)	14(11)	14 (11.5)	NS
*< 4 Cycles chemo first-line*	37 (14.8)	18 (14.2)	19 (15.6)	NS
*ECOG status ≤ 2*	224 (89.9)	118 (92.9)	106 (87)	0.141

Bold values indicate statistically significant

*FIGO* International Federation of Gynecology and Obstetrics, *ECOG* Eastern Cooperative Oncology Group, *NS* not significant

The majority of the patients underwent surgery as part of their treatment: 111 (87.4%) of the patients at center A and 73 (59.8%) of the patients at center B (*p* < 0.001). The vast majority of the patients who never underwent surgery was either because the local team thought no complete clearness could be achieved after review of the images or due to poor performance status or comorbidities of the patients. The main areas of challenge and limitation of operability considered at center B were bowel and mesentery involvement, diaphragmatic/lesser sac and liver capsule involvement, and paracardiac/mediastinal lymph node involvement.

The criteria of inoperability at center A specified mainly extraabdominal nonresectable sites of disease, multiple liver metastases, and poor performance status as per the European Society of Gynaecological Oncology (ESGO)-defined inoperability criteria.^11^

At center A, 77% of the patients had a primary debulking surgery compared with 19.6% of the patients at center B (*p* < 0.001). Consequently, significantly fewer patients had chemotherapy alone without surgery at center A compared with center B (9.4% vs 36%; *p* < 0.001). The number of patients with a new diagnosis who were receiving best supportive care alone did not differ between the two centers (3.1% at center A vs 4.1% at center B).

Combination carboplatin and paclitaxel was used as first-line systemic treatment for the majority of patients at both centers, with only 11% of the patients receiving carboplatin mono. Three-weekly and weekly paclitaxel regimens were not evaluated separately because recent findings show that paclitaxel fractionation does not have an impact on survival.^15^ At both centers, 15% of the patients received less than four cycles of first-line chemotherapy due to reasons such as toxicity, patient’s choice, or disease progression. Also, the two centers did not differ in their rates of maintenance regimens or clinical trial enrollment.

### Intraoperative Tumor Dissemination Patterns and Surgical Morbidity Profile

Intraoperative tumor load was compared between the two centers. A significantly higher median number of tumor-affected IMO fields was reported for the patients at center A than at center B (9 vs 6; *p *< 0.001), as shown in Fig. 1. A significantly higher number of multivisceral resection procedures performed at center A, such as splenectomy (center A, 14.4% vs center B, 0%), pleurectomy (center A, 8.2% vs center B, 0%), diaphragmatic stripping/resection (center A, 57.7% vs center B, 0%), and bowel resection (center A, 65% vs center B, 8.2%), resulted in a significantly higher SCS^8^ at center A than at center B (9 vs 2; *p* < 0.001). This difference was reflected in significantly longer median operation times at center A (285 min) than at center B (155 min) (*p* < 0.001), as well as significantly longer hospital stays at center A (median, 9 days; range, 3–120 days) than at center B (median, 6 days; range, 3–43 days) (*p *< 0.001).

Stoma rates (8.7% in the entire cohort) and postoperative intensive care unit admission rates were similar at the two centers. The number of patients able to receive postoperative chemotherapy did not differ significantly between center A (90%) and center B (97%) (*p* = 0.06). The mean time from surgery to postoperative chemotherapy also did not differ significantly between center A (5.62 weeks; 95% CI 4.6–6.6 weeks) and center B (5.01 weeks; 95% CI 4.1–6.0 weeks).

Total macroscopic tumor clearance was achieved for 84.7% of the patients at center A compared with 58.9% of the patients at center B (*p* < 0.001). The two centers did not differ significantly in terms of major surgical morbidity (11% at both centers), and although a trend toward a higher 28-day mortality was found for the center A cohort, this difference did not reach statistical significance (1.8% vs 0%; *p* = 0.247). Table 2 presents the data in detail.

**Table 2:** Surgical characteristics and tumor dissemination patterns according to the “Intraoperative Mapping of Ovarian Cancer” documentation tool *only* for the patients who underwent cytoreductive surgery

	All patients (*n *= 184) *n* (%)	Center A patients (*n *= 111) *n* (%)	Center B patients (*n *= 73) *n* (%)	*p* value
*Median no. of IMO fields (A1–C3) involved (range)*	8 (0–9)	9 (0–9)	6 (0–9)	**< 0.001**
*Median surgical complexity score (range)*	6 (0–15)	9 (5–15)	2 (0–6)	**< 0.001**
*28 days postop mortality*	2 (1.1)	2 (1.8)	0	0.247
*Median operation time: min (range)*	200 (75–485)	285 (100–485)	155 (75–300)	**< 0.001**
*Residual disease (cm)*				**< 0.001**
None	137 (74.5)	94 (84.7)	43 (58.9)	
0.1–0.5	14 (7.6)	11 (9.9)	3 (4.1)	
0.5–1	14 (7.6)	4 (3.6)	10 (13.7)	
> 2	7 (3.8)	1 (0.9)	6 (8.2)	
*Major surgical morbidity*	18 (10.1)	12 (10.8)	8 (11)	0.8
Reoperation	6 (3.3)	3 (2.8)	3 (4.1)	
Bowel perforation/fistula/leak	4 (2.2)	4 (3.6)	0	
Liver failure	1 (0.5)	1 (0.9)	0	
Renal failure	1 (0.5)	1 (0.9)	0	
DVT/PE	6 (3.3)	5 (4.5)	1 (1.4)	
Secondary wound healing	1 (0.5)	1 (0.9)	0	
Sepsis	5 (2.7)	2 (1.8)	3 (4.1)	
Lymphorrhoea	2 (1)	2 (1.8)	0	
*Procedures performed*				
Splenectomy	16 (8.7)	16 (14.4)	0	
Resection of disease from stomach/lesser sac/celiac trunk	35 (19)	33 (29.7)	2 (2.8)	
Large bowel resection	67 (36.8)	61 (56)	6 (8.2)	
Small bowel resection	31 (16.8)	31(27.9)	0	
Liver/liver capsule resection	48 (26.2)	48 (43.6)	0	
Pleural resection	15 (13.5)	15 (8.2)	0	
Colostomy	12 (6.5)	7 (6.3)	5 (6.8)	
Ileostomy	4 (2.2)	2 (1.8)	2 (2.8)	
Diaphragmatic surgery	64 (34.25)	64 (57.7)	0	

Bold values indicate statistically significant

*IMO* Intraoperative Mapping of Ovarian Cancer, *DVT* deep venous thrombosis, *PE* Pulmonary embolism

Complexity scores are calculated based on the Mayo Clinic algorithm by Aletti et al.^7^

### Follow-Up Evaluation and Survival Data

**Fig. 2:**
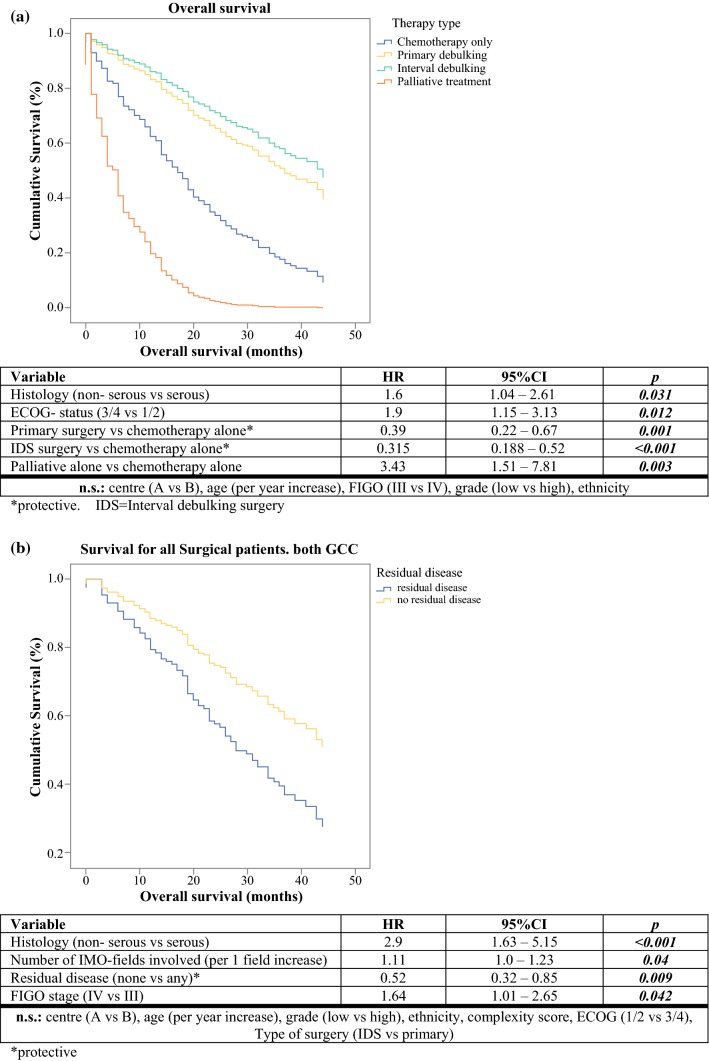
Survival curves and risk factors for mortality. Multivariate analysis (Cox regression) of patients with advanced primary ovarian cancer was performed in two adjacent gynecologic cancer centers. **a** All patients. **b** Surgical patients only (primary and interval surgeries)

*Surgical population:* After a mean follow-up period of 24 months (95% CI 22.18–26.02 months), the mean OS for all the surgical patients was 37.47 months (95% CI 34.43–40.52 months), with a mean OS of 37 months (95% CI 33.17–40.8 months) at center A versus 36.5 months (95% CI 31.8–41.1 months) at center B (*p* = 0.517). Multivariate Cox-regression analysis identified the following factors that negatively affected OS for the surgical cohort: non-serous histology (hazard ratio [HR], 2.9; 95% CI 1.63–5.15), increasing tumor burden as per the number of abdominal fields involved (HR, 1.11 per IMO field; 95% CI 1.0–1.23), and International Federation of Gynecology and Obstetrics (FIGO) stage 4 (HR, 1.64; 95% CI 1.01–2.65). Total macroscopic tumor clearance significantly protected against death (HR, 0.52; 95% CI 0.32–0.85), but treating center and timing of surgery (primary vs interval surgery) had no significant impact. Similarly, age, ethnicity, SCS score, and ECOG status had no significant effect on patients’ survival after surgery. The survival curves are shown in Fig. 2.

*Entire population:* The mean OS for the entire population was 31.87 months (95% CI 29.1–34.62 months). Separately, the entire cohort at center A had a mean OS of 34.37 months (95% CI 30.54–38.19 months), which was significantly higher than at center B: 28.3 months; (95% CI 24.64–31.96; *p* = 0.009). At center B, 64.4% of the patients died during the follow-up period compared with 46% at center A during the same period (*p* = 0.005).

Comparison of the surgical population with the populations that had chemotherapy alone at both centers showed that 43.8% of the patients who had surgery have died versus 86% of the patients who had chemotherapy alone. Multivariate analysis of the entire patient cohort associated a significantly higher risk of death with non-serous histology (HR, 1.6; 95% CI 1.04–2.61), poor ECOG status ≥ 3 (HR, 1.9; 95% CI 1.15–3.13), and supportive care alone (HR, 3.43; 95% CI 1.51–7.81). Cytoreductive surgery of any timing was significantly associated with improved OS compared with chemotherapy alone for both interval surgery (HR, 0.31; 95% CI 0.19–0.52) and primary surgery (HR, 0.39; 95% CI 0.22–0.67). Treatment center, age, FIGO stage (3 vs 4), grade (low vs high), and ethnicity were not prognostically significant (Fig. 2).

## Discussion

Our analysis demonstrated how differing practices and allocations of EOC patients to different treatment pathways can significantly influence their outcome. Debulking also patients with a more extensive tumor dissemination profile and more higher tumor burden requires more resources in terms of theater time and hospital stay. However incorporating maximal-effort debulking surgery into the initial management also of those patients with advanced EOC and high tumor burden, seems to achieve better survival rates even for those unfavorable patients than chemotherapy alone. These findings are consistent with other international published data, which similarly demonstrate that adopting the philosophy of increased surgical effort for higher-stage patients and those with a greater tumor burden results in a significantly more favorable survival over time.^4^ Importantly, although the timing of surgery differed significantly between the two centers, it was not a significant factor in OS.

Although the surgical teams differed between centers A and B, the systemic treatment for many patients was applied by the same medical oncology team at both centers. This was advantageous in this study because it reduced the possibility of subsequent relapse treatments differing enough to influence the comparison of initial intervention and OS.

In the United Kingdom, it is not the gynecologic oncology surgeons who carry out the systemic treatment, but rather the medical oncologists, who often do not treat gynecologic cancers alone. Also, many of the patients from center A opted to have relapse treatment at their local cancer centers and not at center A, so the systemic treatment was not expected to be equally “aggressive,” and hence one could not attribute the more favorable survival in centre A due to a more “aggressive” systemic treatment approach in that centre alone.

The analysis of national UK data collected from EOC patients who received their diagnosis in England between 2008 and 2010 also showed that not receiving any treatment or receiving chemotherapy alone was associated with higher mortality.^16^ However, this of course may well be attributable to selection bias. By including all presenting advanced EOC-patients, not just those who had surgery, in the denominator when evaluating survival, we tried to avoid selection bias, a factor that already has been emphasized as essential for benchmarking and quality assurance elsewhere.^10^,^11^ Similarly, the European Society of Gynaecological Oncology (ESGO) recently defined quality indicators and scores for the surgical management of advanced EOC, also basing them on all presenting patients as the denominator, not only on those who received surgery, in an effort to avoid all selection bias and obtain objective measurements.^11^

Although cytoreductive surgery is a standard part of national and international guidelines,^11^,^13^ in actual practice, a vast proportion of advanced EOC patients will have no surgery at all as part of their initial management. The UK National Cancer Data Repository showed that 44% of patients with newly diagnosed EOC do not receive any type of cytoreductive surgery, whereas 25% receive palliative care alone.^16^ This phenomenon is not unique to the United Kingdom, as no surgery is reported for 21% of the patients in the American National Cancer Database and for 34.2% of the patients in the Surveillance, Epidemiology, and End Results (SEER) database.^17^,^18^ Possible reasons for that trend are apart from patients choice and comorbidities, potentially limited access to specialized care, inability to achieve optimal debulking even after chemotherapy, aversion to surgical complications and higher readmission rates, fear of depleted infrastructural and financial resources, and possible lack of expertise and training.^19^–^21^

At a time when surgeons are judged on the basis of theater times, hospital stay, complications, and readmission rates, it is not surprising that surgeons manifest considerable reluctance to operate on higher risk patients with extensive tumor dissemination that will require more complex surgery.^20^–^23^ A 30-day readmission rate has been internationally proposed as a metric of surgical quality, something that has caused an ongoing clinical concern in the primary treatment of advanced EOC.^22^ With this perspective, Clark et al.^22^ recently conducted a systematic review of all related English literature studies published between 2008 and 2018 to identify risk factors and predictors for 30-day readmission after cytoreductive surgery, making the appeal that policies and programs be designed to measure short- and long-term outcomes in advanced EOC and that bias be avoided in assigning patients to NAC just to maintain low 30-day readmission rates.

Especially when surgery for the patients with a higher tumor load is translated into significantly longer theater times and hospital stays, as we clearly showed in the current analysis, surgeons often are subjected to scrutiny as to why they consider extending their therapeutic effort also to this challenging patient cohort with a presumed less favorable prognosis. A recent survey published by the British Medical Association on challenges in the NHS concerning culture, workforce, and structure^23^ showed that 78% of almost 8000 UK doctors described the NHS resources as inadequate, significantly affecting the quality and safety of patient services, whereas 77% said that national targets and directives are given priority over patient care, resulting in a more defensive and risk-averse practice. Our data, together with the data of multiple others,^4^,^8^ show that by generating a culture of maximal therapeutic effort, even for patients with a higher tumor load, the survival of the entire patient cohort at a center may significantly improve.

A major limitation of the current analysis was the lack of prospective quality-of-life data. Nevertheless, many of the patients who underwent surgery at both centers participated in the international, multicenter, prospective SOCQER 2 study of “patient-reported outcomes after surgery in advanced ovarian cancer”. The preliminary analysis in this study showed no association between surgical complexity and global health status at 12 months, whereas extensive surgery did not seem to cause a decrease in patient quality of life compared with preoperative scores.^9^,^24^ The currently ongoing international Trial of Radical Upfront Surgical Therapy in advanced ovarian cancer (AGO-OVAR-OP.7/NCT02828618) is set to address more of these important quality-of-life questions^25^ and also impact of timing of surgery on overall outcome. Further limitations of the current study were the retrospective design, the rather small sample size and the limited follow-up time.

## Conclusion

The reported findings further support and emphasize the importance of a maximal-effort multimodal approach in advanced EOC, even for patients with greater tumor burden and more extensive tumor spread. These data are important in light of numerous statistics showing that a significant portion of the advanced EOC population is not offered surgery at any point in their journey.^16^–^18^ Finance- and infrastructure-related metrics to evaluate surgical outcome represent a challenge to extend a maximal-effort approach for the patients with advanced disease who require a higher infrastructural support in often-restricted health care systems.^23^

## Additional Information

Because this was a retrospective audit, no individual patient consent was required. The project proposal was reviewed and approved as a clinical audit project by the Quality and Safety Co-ordinators of the Women’s and Children’s Clinical Programme Group at Queen Charlotte’s Hospital, London, and the Mount Vernon–West Hertfordshire Cancer Centre.
